# Cirrhotic Cardiomyopathy: A New Clinical Phenotype

**DOI:** 10.5935/abc.20170066

**Published:** 2017-06

**Authors:** Luis Otávio Cardoso Mocarzel, Mariana Macedo Rossi, Bruna de Mello Miliosse, Pedro Gemal Lanzieri, Ronaldo Altenburg Gismondi

**Affiliations:** Universidade Federal Fluminense (UFF), Rio de Janeiro, RJ - Brazil

**Keywords:** Liver Cirrhosis / mortality, Obesity, Aging, Metabolic Syndrome, Fatty Liver, Cardiomyopathy, Alcoholic

## Introduction

Hepatic cirrhosis is the final spectrum of several aggressions to the liver, with great relevance to public healthcare. National data estimate a prevalence of 0.14% to 0.35%, mortality of 3 to 35 per 100,000 individuals and an annual average of 30,000 hospital admissions in Brazil.^1,2^ With the ageing population, the prevalence of chronic liver diseases, in particular steatohepatitis associated to obesity and metabolic syndrome, results in an increase in the number of hepatic cirrhosis cases.^3^

Cardiac manifestations of hepatic cirrhosis were first reported in the 20^th^ century, with alterations on cardiac output.^4^ With new information on the extra-hepatic repercussions of cirrhosis, cirrhotic cardiomyopathy (CCM) has been described as a spectrum of chronic morphofunctional alterations in the heart of cirrhotic patients with no previous cardiac diseases.^5-7^ The cardiomyocyte lesion is provoked by an imbalance in hemeostasis that occurs in the progression of cirrhosis, with exhaustion of beta-adrenergic receptors, cytoplasmic impregnation by endocannabinoids, and imbalance of nitric oxide and endothelin.^7^ CCM is asymptomatic; however, systolic and diastolic structural alterations are described in the electrocardiogram (ECG) and Doppler echocardiogram (ECHO).^8^

Because CCM is asymptomatic, except during situations of stress, prevalence studies are limited. Heart failure (HF) secondary to CCM is frequent in patients who undergo liver transplant, in which half the patients presents HF, and up to 21% die from cardiac causes.^9^ Today, it is possible to identify myocardial compromise in up to 50% of cirrhosis patients,^10^ but, in most cases, without clinical expression.

The objective of this review is to describe recent findings of the pathophysiology of the cardiovascular system in hepatic cirrhosis, and show the importance of biomarkers and cardiac imaging methods in the identification of a new clinical phenotype of CCM.

### Cardiovascular system in hepatic cirrhosis

Hepatic cirrhosis evolution is insidious, being at times asymptomatic or oligosymptomatic until advanced stages. Signs and symptoms of liver failure tend to appear late, with subtle clinical and laboratory manifestations which are often hard to interpret.

Cardiologists may be faced with a patient complaining of dyspnea, presenting with ascites, without pathological jugular swelling, normal ECG, ECHO with normal ejection fraction, but with elevated B-type natriuretic peptide (BNP) - a condition that may be suggestive of CCM.^11^ Considering it is different from a classic presentation of HF, it is necessary to know this syndrome (CCM) and have a degree of clinical suspicion for early identification, to prevent its evolution to related complications, such as suprarenal insufficiency and hepatorenal syndrome (HRS).

In the past, cardiomyopathy in alcoholic cirrhosis was understood as myocardial damage concomitant to liver damage, and had dilated cardiomyopathy as a phenotype.

**Figure 1:**
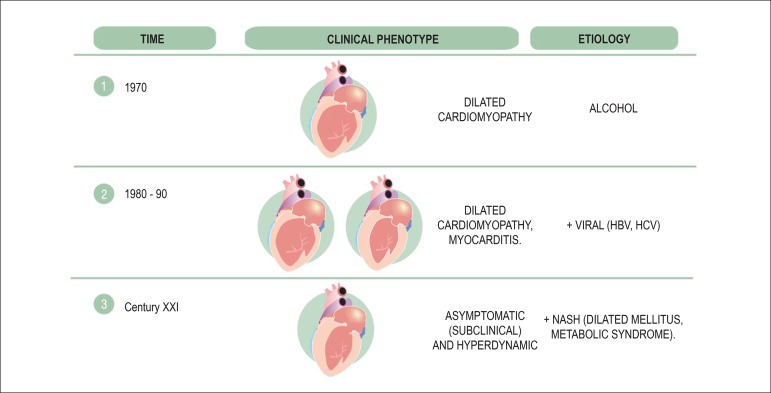
Evolution of cirrhotic cardiomyopathy concept. HBV: hepatitis B virus; HCV: hepatitis C virus; NASH: non-alcoholic steatohepatitis.

It was believed that alcohol aggression to the heart always happened in the form of chronic disease with dilatation of cavities. With the discovery of viral hepatitis B and C, myocarditis from hepatitis B and C viruses was described, with variable clinical phenotypes, from the oligosymptomatic state, associated or not to dilated cardiomyopathy. The concept of CCM allows us to understand a new clinical phenotype: the asymptomatic patient, with no apparent functional limitations, but subclinical cellular and structural cardiac disease (Figure 1).

Cirrhotic patients have hyperdynamic circulation from the peripheral vasodilatation imposed by the neuroendocrine imbalance of hepatic cirrhosis, with increased cardiac output at rest and decreased peripheral vascular resistance.^12^ There is a predominance of arterial vasodilatation, which induces the activation of the autonomic nervous and renin-angiotensin-aldosterone (RAAS) systems, so that peripheral perfusion is preserved. This hyperdynamic pattern is directly dependent on cardiac reserve (inotropic and chronotropic capacity), so cardiac output is preserved.

**Figure 2:**
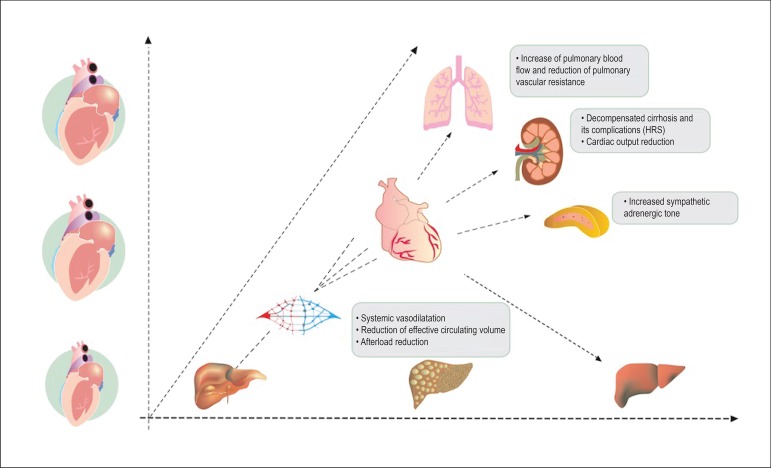
The progression of cardiac disease is concomitant to its evolution to hepatic cirrhosis, evolving from diastolic dysfunction, systolic dysfunction, and dilated cardiomyopathy. HRS: hepatorenal syndrome.

Figure 2 summarizes the main hemodynamic alterations in cirrhotic patients. There is a relative increase in cardiac output, sympathetic hyperstimulation, and elevation of heart rate and pulmonary blood flow, with a reduction of pulmonary vascular resistance. Conversely, there is a decrease in effective circulating arterial volume, systemic blood pressure, and afterload from vasodilatation.^12^

With the evolution of biochemical studies and morphofunctional cardiac evaluations, the concept of CCM started to represent the suboptimal ventricular response to stress, physiological or induced, even though the patient presents apparently normal cardiac output at rest, in the absence of previous heart disease.^13^

CMC`s pathogenesis involves cellular, neural and humoral factors, whose pathophysiological basis is in alterations of the plasma membrane of cardiomyocytes: influence in calcium signaling, hyperstimulation of beta receptors, action mediated by nitric oxide, carbon monoxide and endocannabinoids. There is an increase in circulating levels of vasoactive substances (endothelin, glucagon, vasoactive intestinal peptide, tumor necrosis factor, prostacyclins, and natriuretic peptide) which are usually elevated in cirrhosis due to liver failure and the presence of portosystemic collateral vessels.^14^

Concomitant to the progression of the hepatic disease, there is diastolic myocardial dysfunction (myocardial rigidity due to fibroses, myocardial hypertrophy and subendothelial edema) and systolic myocardial dysfunction (hyperdynamic circulation and splanchnic vasodilation, with increased arterial compliance).^14^

It is understood that diastolic and systolic dysfunction is directly related to the severity of liver dysfunction and portal hypertension.^5^ Diastolic dysfunction usually precedes systolic dysfunction, which is generally observed in situations in which there is an increased demand for cardiac output associated to decreased myocardial contractility, such as in situations of hemodynamic stress - infectious processes, physical exercise, use of certain medication and surgery.^9^

Cardiac dysfunction can negatively interfere in the prognosis of cirrhotic patients, reducing survival and participating in the genesis of complications. HRS and post-paracentesis circulatory dysfunction, which a state of systemic hypoperfusion secondary to the quick removal of large volumes of ascitic fluid without adequate albumin intake, are the main complications associated to blunt myocardial response to stress.^15^ Cardiac dysfunction is also manifested in situations of myocardial stress, such as preload increase secondary to transjugular intrahepatic portosystemic shunt (TIPS) insertion, generally indicated for pre liver-transplant patients.^10^

### CCM diagnosis approach

Considering most patients are asymptomatic in the initial stages of CCM, they must undergo clinical, laboratory, electrocardiographic and imaging evaluations for early diagnosis.^11,16^ Criteria for CCM identification are described in Table 1.

**Table 1:** Diagnostic criteria for cirrhotic cardiomyopathy

**Clinical-laboratory criteria**	
Absence of cardiopulmonary symptoms at rest	
Low functional cardiac reserve	
Signs of sympathetic hyperactivity and RAAS	
El BNP, pro-BNP and/or troponin elevation	
Electrocardiographic criterion	
QT interval prolongation	
**Echocardiographic criteria**	
Diastolic dysfunction	
**E/A ratio < 1.0**	
Left atrial enlargement	
Deceleration time > 200 ms	
Isovolumetric relaxation time > 80 ms	
Increased left ventricular end-diastolic diameter	
Left ventricular hypertrophy	
Systolic dysfunction	
Left ventricular function at rest below 55%	
Contractility deficit in situations of overload (stress)	

RAAS: renin-angiotensin-aldosterone system; BNP: B-type natriuretic peptide. (*) Criteria that corroborate the diagnosis of CCM according to the World Congress of Gastroenterology in Montreal, Canada, 2005.

The use of biomarkers has been useful in clinical practice, especially troponin I, BNP, and N-terminal-pro-BNP (NT-pro-BNP), which may be found in abnormal levels in cirrhosis.^16-18^ Troponin I elevation has been associated to a decrease in systolic output and left ventricular mass, but with no correlation to the severity of cihrrosis.^19^ Pro-BNP elevation has been associated to intraventricular septum wall thickness and ventricular wall thickness. BNP and pro-BNP elevation is associated to the severity of cirrhosis and cardiac dysfunction, but not to hyperdynamic cuiculation.^20,21^ The increase of BNP and pro-BNP in cirrhotic patients, compared to the control group and healthy individuals, has a direct correlation to the severity of the hepatic disease (by the Child-Pugh score and the hepatic venous pressure gradient) and to cardiac dysfunction markers (QT interval, heart rate, and plasma volume).^16^ Elevated levels of BNP and pro-BNP in cirrhotic patients indicate a myocardial origin of these peptides due to the stretching of cardiomyocytes from left ventricular overload, which increases the expression of the gene responsible for BNP transcription.^17^

Tumor necrosis factor alpha and interleukins 1 and 6 are inflammatory cytokines hyperstimulated in hepatic cirrhosis and HF. The elevation of cardiac dysfunction biomarkers (troponin I, BNP, and pro-BNP) indicates, in the context of cirrhosis, myocardial compromise, which is related to the severity of the hepatic disease.^16^

Chest X-Ray evaluation is usually normal, or may reveal indirect signs of left atrial enlargement and, in advanced stages, left ventricular enlargement and cardiomegaly with pleural effusion. ECG may aid in the diagnosis by showing QT interval prolongation (earliest and most prevalent alterations), presence of multiple extrasystoles and, in more advanced stages, bundle branch block and ST segment depression. 24-hour holter has better sensitivity to identify bradyarrhythmia and tachyarrhythmia, and can aid in the diagnosis of subclinical or paroxysmal diseases.

ECHO is a non-invasive method whose findings are correlated to the degree of hepatic dysfunction: increase of LV diastolic diameter and decrease of peak systolic velocity, and LV systolic deformity rate evaluated by tissue Doppler. Other findings seen in CCM's diastolic dysfunction are: reduced early (E) and late (A) ventricular relaxation capacity, and decreased E/A ratio with prolongation of the E-wave deceleration time. In advanced stages, there is LV systolic dysfunction, with reduction of the ejection fraction. The strain rate (SR) is a new echocardiographic parameter able to identify a reduction in LV systolic function when the ejection fraction is still normal.^5^

MRIs have been increasingly used in the context of morphofunctional evaluation of liver and heart diseases. It can determine ejection fraction, volume of cardiac chambers (increase of LV mass and end diastolic volumes in the LA and LV) and myocardial morphologic alterations, including tissue alterations (areas of edema and fibrosis), identifying the lesion by using contrast such as gadolinium.^8^ It can also help identify simultaneous compromise of both organs, such as in hemochromatosis and amyloidosis.

Recognizing the appropriate moment for a therapeutic approach in these patients is a challenge in the comprehension of CCM. Cardiac compromise is usually subclinical, and is manifested as left ventricular insufficiency (LVI) at times of increased demand, such as in situations of clinical or surgical stress. Congestive HF, with signs of pulmonary congestion, is the final spectrum of dilated CMPs of any etiology, in which CCM is included - clinical context of poor prognosis and high mortality. There is still no specific treatment for CCM. It is currently approached in the same way as HF, which includes water and sodium restriction, use of diuretics, RAAS inhibitors and beta-blockers.^19^

CCM approach in the course of hepatic cirrhosis is still a challenge in clinical practice because, when there is the diagnosis of dilated CMP with frank pulmonary congestion, prognosis is reserved. Recently, our group reported, for the first time, two cases of patients with elevated BNP, X-Ray with no pulmonary congestion, and ECHO with normal LVEF, but with a progression to HRS refractory to conventional treatment, in which there was benefit from the use of dobutamine, as rescue therapy of kidney function, with great clinical response.^22,23^ The central idea is that HRS is a marker of bad systemic perfusion, and that cardiac output, despite being in the normal range in the ECHO, is insufficient for the demand. Thus, the inotropic would promote an increase in cardiac output and renal perfusion. In the published cases, there was good clinical response with recovery of kidney function.

## Conclusion

Myocardial compromise, underdiagnosed in cirrhotic patients, and CCM represent a new clinical phenotype. Once cardiovascular repercussions are understood, the cardiologist should observe its manifestations, be them signs of congestion or clinical complications such as HRS, particularly in situations of clinical or surgical stress, stimulating its evaluation with cardiac imaging methods and biomarkers. There is still a lack of understanding of how to apply this knowledge, in daily practice, to benefit patients. There is a need for studies with the objective of identifying potential treatments that alter the natural history of cardiac disease in cirrhotic patients, especially in the asymptomatic phase.
